# Nuclear Ras2-GTP Controls Invasive Growth in *Saccharomyces cerevisiae*

**DOI:** 10.1371/journal.pone.0079274

**Published:** 2013-11-14

**Authors:** Serena Broggi, Enzo Martegani, Sonia Colombo

**Affiliations:** 1 Department of Biotechnology and Biosciences, University of Milano-Bicocca, Milan, Italy; 2 SysBio Centre of Systems Biology, Milan, Italy; II Università di Napoli, Italy

## Abstract

Using an eGFP-RBD3 probe, which specifically binds Ras-GTP, we recently showed that the fluorescent probe was localized to the plasma membrane and to the nucleus in wild type cells growing exponentially on glucose medium, indicating the presence of active Ras in these cellular compartments. To investigate the nuclear function of Ras-GTP, we generated a strain where Ras2 is fused to the nuclear export signal (NES) from the HIV virus, in order to exclude this protein from the nucleus. Our results show that nuclear active Ras2 is required for invasive growth development in haploid yeast, while the expression of the NES-Ras2 protein does not cause growth defects either on fermentable or non-fermentable carbon sources and does not influence protein kinase A (PKA) activity related phenotypes analysed. Moreover, we show that the cAMP/PKA pathway controls invasive growth influencing the localization of active Ras. In particular, we show that PKA activity plays a role in the localization of active Ras and influences the ability of the cells to invade the agar: high PKA activity leads to a predominant nuclear accumulation of active Ras and induces invasive growth, while low PKA activity leads to plasma membrane localization of active Ras and to a defective invasive growth phenotype.

## Introduction

In the yeast *Saccharomyces cerevisiae* the Ras/cAMP/PKA signaling pathway plays an important role in the control of metabolism, stress resistance, proliferation [1], [2], [3], [4] and it also affects morphogenesis and development, including pseudohyphal, invasive growth and sporulation [5]. The cAMP-dependent protein kinase A (PKA) is a central component of this pathway. PKA is a tetramer consisting of two regulatory subunits (encoded by *BCY1*) and two catalytic subunits (encoded by *TPK1*, *TPK2* and *TPK3*). PKA activity depends on cAMP synthesized by adenylate cyclase (encoded by *CYR1*), which is activated by the Ras proteins and the GPCR system [6]. Binding of cAMP to the PKA regulatory subunit results in dissociation and thereby activation of the catalytic subunits, which phosphorylate a variety of proteins. Mutants with attenuated PKA activity display hyperaccumulation of cAMP, whereas mutants with an hyperactivated PKA pathway display reduced cAMP level. Data in literature show that PKA regulates the localization of cAMP/PKA signaling pathway components, like the *PDE2*-encoded high-affinity cAMP phosphodiesterase [7], Cdc25 [8] and the Ras2 protein [9]. The Ras proteins were highly conserved during evolution and they function as a point of convergence for different signalling pathways in eukaryotes. Recent reports have more and more emphasized the importance of subcellular compartmentalisation in small GTPases signalling [10]. Recently, to investigate the localization of active Ras *in vivo* in *S. cerevisiae* we used a probe (eGFP-RBD3) expressing eGFP fused to three sequential Ras Binding Domains of Raf1, which binds Ras-GTP with a much higher affinity than Ras-GDP [11], [12], [13]. We observed that the eGFP-RBD3 probe was localized to the plasma membrane in wild type cells growing exponentially on glucose medium, indicating the presence of active Ras in this cellular compartment. Surprisingly the probe was also found to accumulate within the nucleus and only marginally in mitochondria. Active Ras is not the only component of the cAMP/PKA pathway found to be localized in the nucleus, since PKA was found almost exclusively nuclear in glucose growing cells, whereas in respiring or in stationary phase cells, Bcy1 and Tpk1 were more evenly distributed over both nuclear and cytoplasmic compartments [14]. Also Cdc25 and Ira1 were recently found to accumulate in the nucleus, while Cyr1 was not found in this compartment, but it was mainly localized in internal membranes [8], [15]. A physiological purpose of the nuclear localization of active Ras2 could be to separate the protein from adenylate cyclase, preventing overstimulation of cAMP synthesis. On the other end, it could be indicative of a specific nuclear function of Ras-GTP. More specifically, Ras2, but not Ras1, activates invasive growth using either of two downstream signalling pathways, the filamentation MAPK cascade (Cdc42p/Ste20p/MAPK) or the cAMP/PKA pathway, indicating a crosstalk between both signalling pathways and this could happen in the nucleus [16], [17], [18]. This hypothesis is substantiated by the observation that in a strain deleted in *GPA2*, which has a defect in pseudohyphal growth [19], active Ras does not accumulate in the nucleus, but is mainly localized in mitochondria [13].

In this paper we show that the localization of active Ras is dependent on PKA activity. In particular, in a strain with absent PKA activity (*cyr1Δ pde2Δ yak1Δ* strain) more then 70% of the cells show a plasma membrane localization of active Ras, while in a strain with high PKA activity (*bcy1*Δ strain), Ras-GTP is localized mainly in the nucleus. To investigate the nuclear function of Ras2, we generated strains where this GTPase is fused to the nuclear export signal from the HIV virus (HIV virus Rev protein NES) [20], in order to completely exclude it from the nucleus. Our results show that cells lacking active Ras2 in the nucleus are not capable of invasive growth, while the ability to grow either on fermentable or non-fermentable carbon sources is not impaired, as well as sensitivity to heat shock, osmotic and oxidative stress. Finally, we show that PKA activity controls invasive growth influencing the localization of active Ras proteins, since cells with high PKA activity, showing a predominant nuclear accumulation of active Ras, are able to invade the agar medium, while a mutant strain with absent PKA activity lost this ability.

## Materials and Methods

### Yeast Strains and Growth Conditions

*Saccharomyces cerevisiae* strains used in this study are listed in Table 1. Rich medium (YP) contained 1% yeast extract (Biolife), 2% tryptone (Biolife), 2% agar (Biolife) and was supplemented with 2% glucose (YPD), 3% ethanol (YPE) or 3% glycerol (YPGly) as carbon source. Synthetic complete media (SD) containing 2% glucose, 6.7 g/l YNB w/o aminoacids (Becton and Dickinson Italia, Buccinasco) and the proper selective drop-out CSM (Complete Synthetic Medium, supplied by BIO101, California, USA) were used for growth kinetics and immunofluorescence experiments. The cell density of liquid cultures grown at 30°C was determined with a Coulter Counter Z2 (Beckman Coulter, Cassina de Pecchi) on mildly sonicated, diluted samples.

**Table 1:** Yeast strains used in this study.

Strains	Genotype	Source/Ref.
W303-1A	*MATa ade2-1 can1-100 his3-11,15 leu2-3112 trp1-1 ura3-1*	Thomas and Rothstein (1989) [21]
W303 *cdc35 pde2 yak1*	*MATa* W303-1A *cdc35::KanMX pde2::TRP1 yak1::LEU2*	Görner *et al*. (2002) [22]
SP1	*MATa his3 leu2 ura3 trp1 ade8 can1*	Nikawa *et al*. (1987) [23]
S18-1D	*MATα SP1 tpk1* ^w1^ *tpk2::HIS3 tpk3::TRP1*	Nikawa *et al*. (1987) [23]
SGP406	*MATa SP1 tpkl*::*URA3 tpk2*::*HIS3 tpk3*::*TRPl yakl*::*LEU2*	Garrett *et al*. (1989) [24]
*JW1*	*MATa* W303-1A *bcy1::URA3*	Griffioen *et al*. (2000) [14]
*GG104*	*MATa* W303-1A *cdc35::KanMX pde2::TRP1 msn2::HIS3 msn4::TRP1*	Roosen *et al*. (2005) [25]
PM731	*MATa* W303-1A *gpa2::URA3*	Colombo *et al.* (1998) [26]
Tlys86	*MAT*a *leu2::hisG ura3-52 trp1::hisG*	Magherini *et al.*(2006) [27]
W303-1A-NES-Ras2	W303-1A *ras2*::NESRAS2-SpHIS5	This work
Tlys86-NES-Ras2	Tlys86 *ras2*::NESRAS2-URA3	This work

### Plasmids

To generate the W303-1A-NES-RAS2 strain we used a plasmid-derived gene replacement cassettes, which was obtained with the following strategy. The *RAS2* coding sequence was amplified by PCR using the YCp50RAS2 plasmid as template and the following primers: forward primer containing the *Eco*RI restriction site (sequence underlined) CCGgaattc **ATG**CCTTTGAACAAGTCGAAC and reverse primer containing the *Sac*I restriction site (sequence underlined) CGCgagctc **TTA**ACTTATAATACAACAGCCACCCG. The amplified sequence was then purified using the JETSORB Gel Extraction Kit (Genomed GmbH, Lönne, Germany), digested with *Eco*RI and *Sac*I and ligated with the pNESHIS5 construct [15], carrying the HIV Rev protein nuclear export sequence, previously digested with the same restriction enzymes. The pNESRAS2 construct obtained was used as template to amplify the *RAS2* gene replacement cassettes, using the following primers: forward primer TAAAAAAACCAAGTTAACCGTTTTCGAATTGAAAGGAGATATACAGAAAAAAAA **ATGACCATGATTACGCCAAG** (underlined: nucleotides −1 −54 upstream the *RAS2* ATG start codon; in bold: nucleotides 1–20 of the pNESRAS2 plasmid) and reverse primer CCGGCAACCATATGATATTGCCCAAAGTTTCCAATGTTAACAAGAATGAAACGG **CGCCACTTCTAAATAAGCGA** (underlined: nucleotides +138+191 downstream the *RAS2* TAA stop codon; in bold: nucleotides 2614–2633 of the pNESRAS2 plasmid). Finally, the cassette was used to transform strain W303-1A and transformants were selected on – histidine selective plates.

To generate the Tlys86-NES-RAS2 strain we used a plasmid-derived gene replacement cassettes, which was obtained with the following strategy. The pNESRAS2 construct was digested with *Hpa*I (cutting upstream NES) and *Sac*I (cutting downstream *RAS2*); the fragment containing the fusion NES-RAS2 was purified using the JETSORB Gel Extraction Kit and ligated with pyx212 (ingenius) digested with *Sma*I and *Sac*I. The pyx212NESRAS2 construct obtained was used as template to amplify the *RAS2* gene replacement cassettes, using the following primers: forward primer TAAAAAAACCAAGTTAACCGTTTTCGAATTGAAAGGAGATATACAGAAAAAAAA **ATGACCATGATTACGCCAAG** (underlined: nucleotides −1 −54 upstream the *RAS2* ATG start codon; in bold: nucleotides 1–20 of the pNESRAS2 plasmid) and reverse primer CCGGCAACCATATGATATTGCCCAAAGTTTCCAATGTTAACAAGAATGAAACGG **TACTGAGAGTGCACCATACC** (underlined: nucleotides +138+191 downstream the *RAS2* TAA stop codon; in bold: nucleotides 3023–3042 of the pyx212NESRAS2 construct). Finally, the cassettes was used to transform strain Tly86 and transformants were selected on – uracil selective plates.

Plasmids pYX212-eGFP-RBD3 and pYX242-eGFP-RBD3 have been previously published [12]; [13].

### Fluorescence Microscopy

Cells were grown at 30°C until exponential phase. Subsequently, 40 µl of cells suspension were seeded on concanavalin A (Sigma-Aldrich, Milano, Italy)-coated cover glass for 10 min (100 µg/ml). The cover glass was washed 4 time using the proper medium and put on top of a Thoma chamber. Images were acquired with a Nikon Eclipse E600 microscope equipped with a 60×, 1.4 oil Plan-Apochromatic objective and a standard FITC filter set (Nikon, EX 450–490, DM 505, BA 520) for GFP fluorescence. Images were recorded digitally using a Leica DC 350F camera and processed using Adobe Photoshop (Adobe Systems, Inc.).

### Determination of Ras2-GTP/total Ras2 Ratio *in vivo*

Determination of the ratio Ras2-GTP/total Ras2 was performed essentially as described previously [28], [29]. This assay exploits the known specificity of the interaction between Ras-GTP and the RBD of Raf-1 to detect activated Ras. Cells were grown in glucose medium till exponential phase. At time 0, KOH was added to raise the pH to 8.0 (12.5 mM, final concentration) and samples were collected by filtration on nitrocellulose filters. After the addition of ice-cold lysis buffer [25 mM HEPES, pH 7.5, 150 mMNaCl, 1% (w/v) Nonidet P-40, 0.25% (w/v) sodium deoxycholate, 10% (w/v) glycerol, 25 mM NaF, 10 mM MgCl2, 1 mM EDTA, 1 mM sodium vanadate, one tablet of Protease Inhibitor Mixture (Roche Applied Science) in 50 mL of extraction medium], cells were disrupted with glass beads in a Fastprep instrument. Cleared supernatant (containing 200 µg of total protein) was incubated with 20 µL of bed volume of glutathione S-transferase (GST)-RBD fusion protein prebound to glutathione-Sepharose for 1 h at 4°C. Bound proteins were eluted with 1.25× sodium dodecyl sulphate (SDS)-sample buffer [100 mM Tris-HCl, pH 6.8, 2% (w/v) β-mercaptoethanol, 4% (w/v) SDS, 0.2% (w/v) bromophenol blue, 20% (w/v) glycerol], separated by SDS-PAGE (polyacrylamide gel electrophoresis), blotted onto nitrocellulose, immunodecorated with anti-Ras2 polyclonal antibodies (SC-6759, Santa Cruz Biotechnology), and revealed with an ECL Western blotting analysis system (Genespin). Total Ras2 protein was detected in cleared lysate by Western blotting using the same anti-Ras2 antibodies. The ratios of Ras2-GTP/total Ras2 were determined by densitometric analysis (SCIONIMAGE software).

### Invasive Growth Assay

5 µl of an overnight saturated culture of the indicated strains of the W303-1A background, grown in YPD at 30°C, were spotted onto a YPD plate and grown at the same temperature for 3 days. Total growth of strains was imaged and then the plate was gently washed with water, from the top down, with a Pasteur pipette to remove non-invasive cells from the surface of the agar. The plates were allowed to dry briefly before re-imaging to document invasive growth. For the indicated strains of the Tly86 background, 10 µl of an overnight saturated culture were spotted onto a YPD plate and after 3 days at 30°C the plate was washed under the running water. Pictures were taken before (Total growth) and after (Invasive growth) washing.

### Heat-shock Sensitivity Assay

For the heat-shock sensitivity assay in liquid medium, cells were grown in synthetic complete medium containing 2% glucose at 30°C to mid-exponential growth phase (about 5×10^6^ cells/ml), then diluted in fresh medium to a concentration of 1.25×10^6^ cells/ml. Cells were then exposed to 51°C for the indicated time (min) and then 5 µl (6×10^3^ cells) were spotted onto YPD agar plates and incubated at 30°C for 24 hours.

### Osmotic and Oxidative Stress Sensitivity Assay

For the osmotic and oxidative stress sensitivity assay in liquid medium, cells were grown in YPD at 30°C to mid-exponential growth phase (about 5×10^6^ cells/ml), harvested by centrifugation, washed 3 times with sterile water and resuspended in sterile water at 10^7^ cells/ml. 5 µl from the concentrated suspension and from three 10–fold dilutions were spotted on YPD agar plates containing 2 mM H_2_O_2_, 6 mM H_2_O_2_ or 0.5 M NaCl. Plates were incubated at 30°C for 2 days.

### Growth on Different Carbon Sources

To test growth on different carbon sources, cells were grown in YPD at 30°C to mid-exponential growth phase (about 5×10^6^ cells/ml), harvested by centrifugation, washed 3 times with sterile water and resuspended in sterile water at 10^7^ cells/ml. 5 µl from the concentrated suspension and from three 10–fold dilutions were spotted on YP agar plates containing 2% glucose (YPD), 3% glycerol (YPGly) or 3% Ethanol (YPE). Plates were incubated at 30°C for 2 days.

## Results

### PKA Regulates the Localization of Active Ras

**Figure 1:**
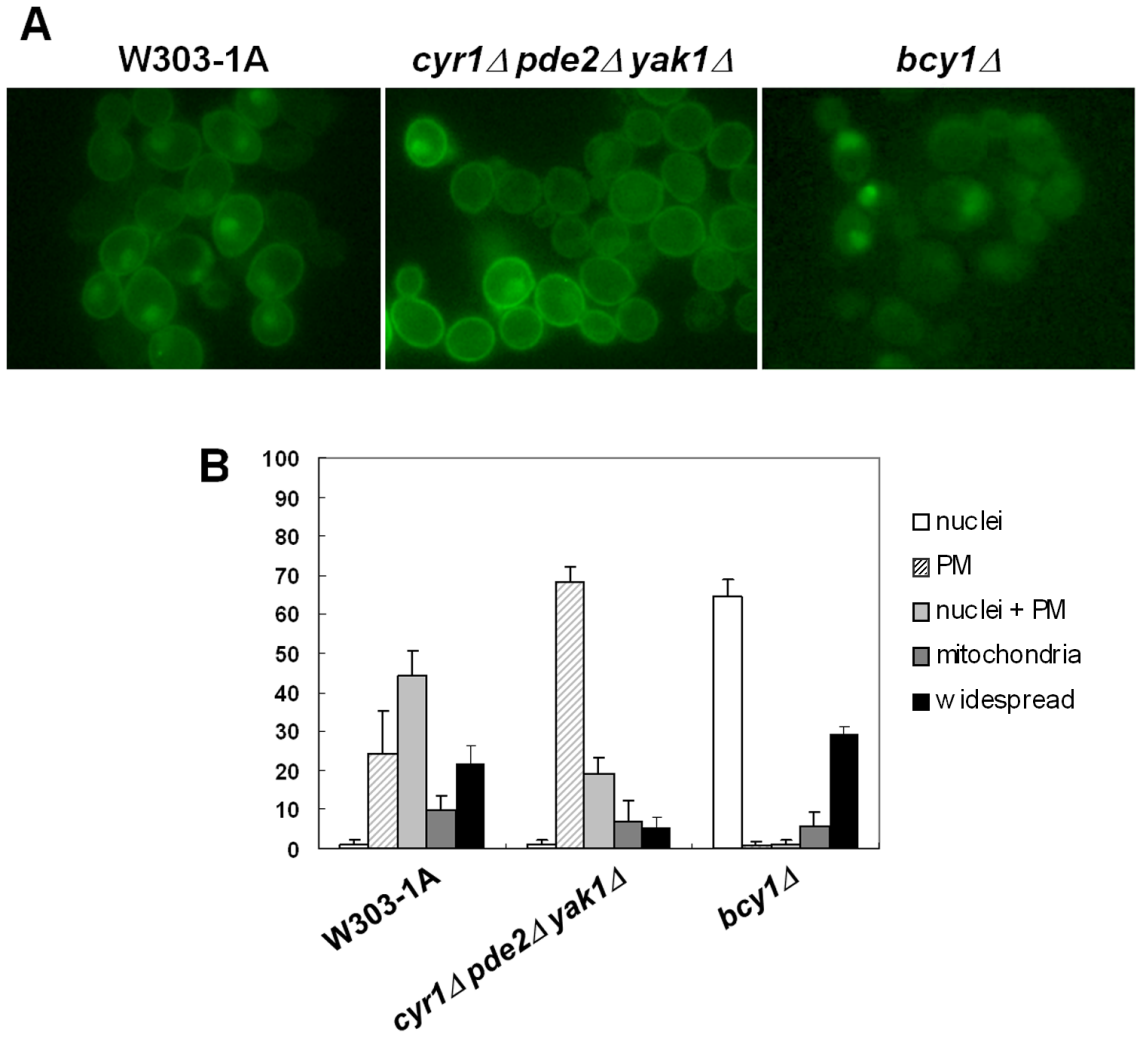
Effect of PKA activity on the localization of active Ras. (A) W303-1A, *cyr1*Δ *pde2*Δ*yak1*Δ and *bcy1*Δ cells transformed with YEpeGFP-RBD3. Cells were grown in medium containing 2% glucose at 30°C until exponential phase and then photographed with a Nikon fluorescence microscope. (B) Subcellular distribution of eGFP fluorescence.

It has been shown that PKA regulates the localization of cAMP/PKA signaling pathway components, like the *PDE2*-encoded high-affinity cAMP phosphodiesterase [7], Cdc25 [8] and the Ras2 protein [9]. To investigate whether PKA activity plays a role in the localization of active Ras, we introduced the eGFP-RBD3 probe, which binds specifically to Ras-GTP [12], [13], into the *cyr1Δ pde2Δ yak1Δ* strain [22], bearing a deletion in the gene encoding adenylate cyclase. Deletion of *PDE2* allows this strain to use cAMP added to the medium, bypassing lethality caused by deletion of *CYR1* [30], [31], while deletion of *YAK1* allows this strain to grow even in the absence of cAMP [22], [24]. Our results showed that in this strain, active Ras was predominantly membrane localized in cells growing on synthetic complete medium containing 2% glucose (Figure 1). A comparable result was obtained using a strain with either reduced PKA activity (*tpk1* ^w1^ *tpk2*Δ *tpk3*Δ) or absent PKA activity (*tpk1Δ tpk2Δ tpk3Δ yak1Δ*) (Figure S1). We have also examined the localization of active Ras proteins in the *bcy1Δ* strain, lacking the PKA regulatory subunit gene and showing elevated and constitutive PKA activity. In this strain, Ras-GTP was found to be mainly localized in the nucleus, while no active Ras was localized at the plasma membrane anymore (Figure 1). As previously reported [13], in wild type cells, active Ras accumulated mainly at the plasma membrane and in the nucleus during growth on medium containing glucose (Figure 1).

The data presented here clearly show that the localization of active Ras is actually dependent on PKA activity. In particular, in a strain with high PKA activity no active Ras is localized at the plasma membrane, while in a strain with absent PKA activity more then 70% of the cells show a strong plasma membrane localization of active Ras, while the nuclear localization is dramatically reduced compared with the wild type strain (Figure 1B).

**Figure 2:**
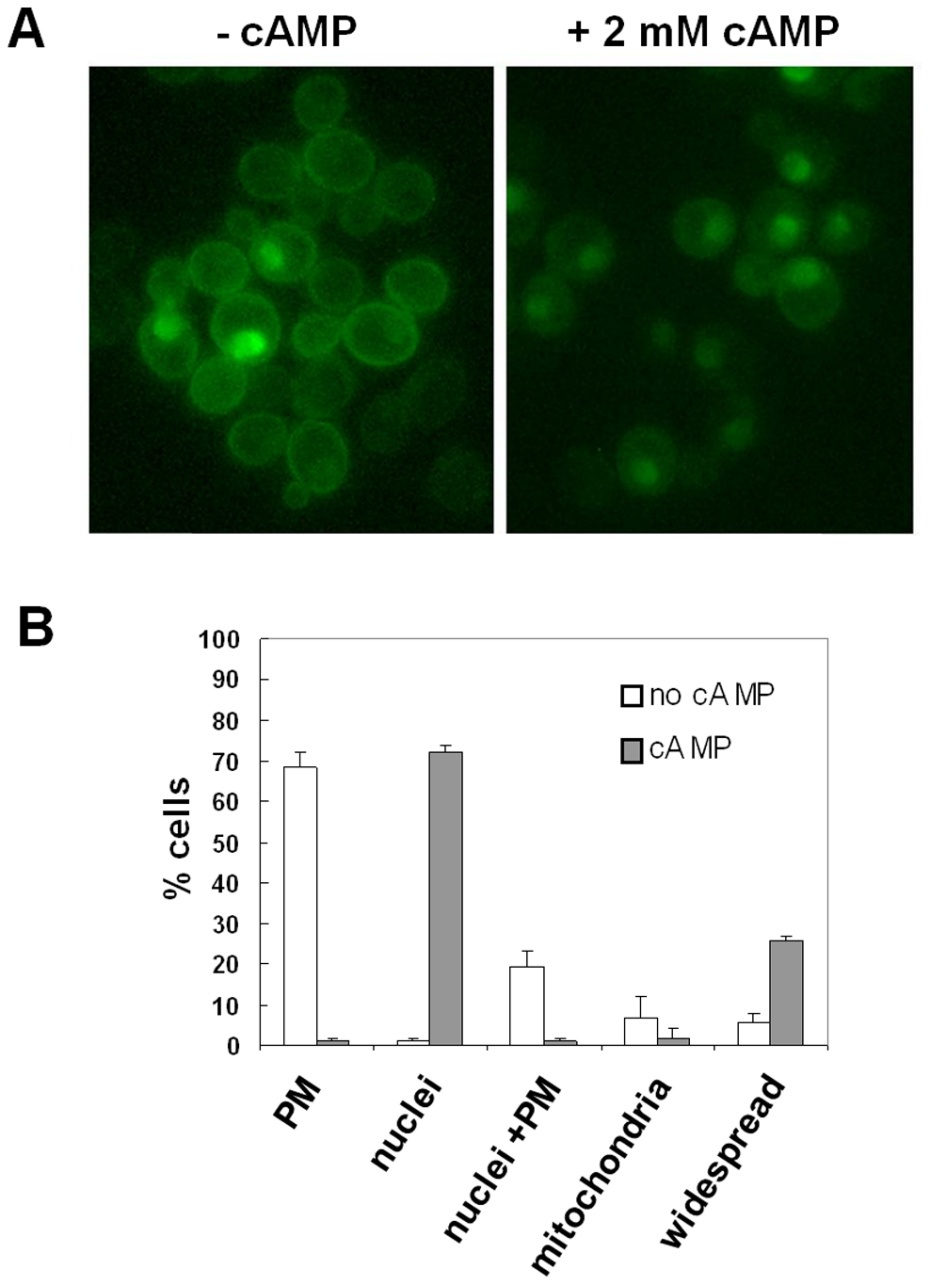
Localization of active Ras in glucose-growing *cyr1Δ pde2Δ yak1Δ* cells, before and after addition of cAMP. (A) *cyr1Δ pde2Δ yak1Δ* cells transformed with YEpeGFP-RBD3 were grown in medium containing 2% glucose at 30°C until exponential phase and then photographed with a Nikon fluorescence microscope, before and 45 min after addition of 2 mM cAMP. (B) Subcellular distribution of eGFP fluorescence.

To better investigate the effect of PKA hyperactivation on active Ras localization, we examined the localization of the probe in the *cyr1Δ pde2Δ yak1Δ* strain, before and after addition of 2 mM cAMP. In this strain, PKA activity is not required for growth, but PKA can be activated by addition of exogenous cAMP. Cells were grown on glucose medium until exponential phase and then 2 mM cAMP was added directly to the cell cultures. We observed that 45 minutes after addition of cAMP, the probe localized mainly in the nucleus (Figure 2), supporting the idea that the localization of active Ras is influenced by PKA activity and in particular that high PKA activity promotes the localization of the probe from the plasma membrane to the nucleus. A comparable result was obtained using the *cyr1Δ pde2Δ msn2Δ msn4Δ* strain (Figure S2).

**Figure 3:**
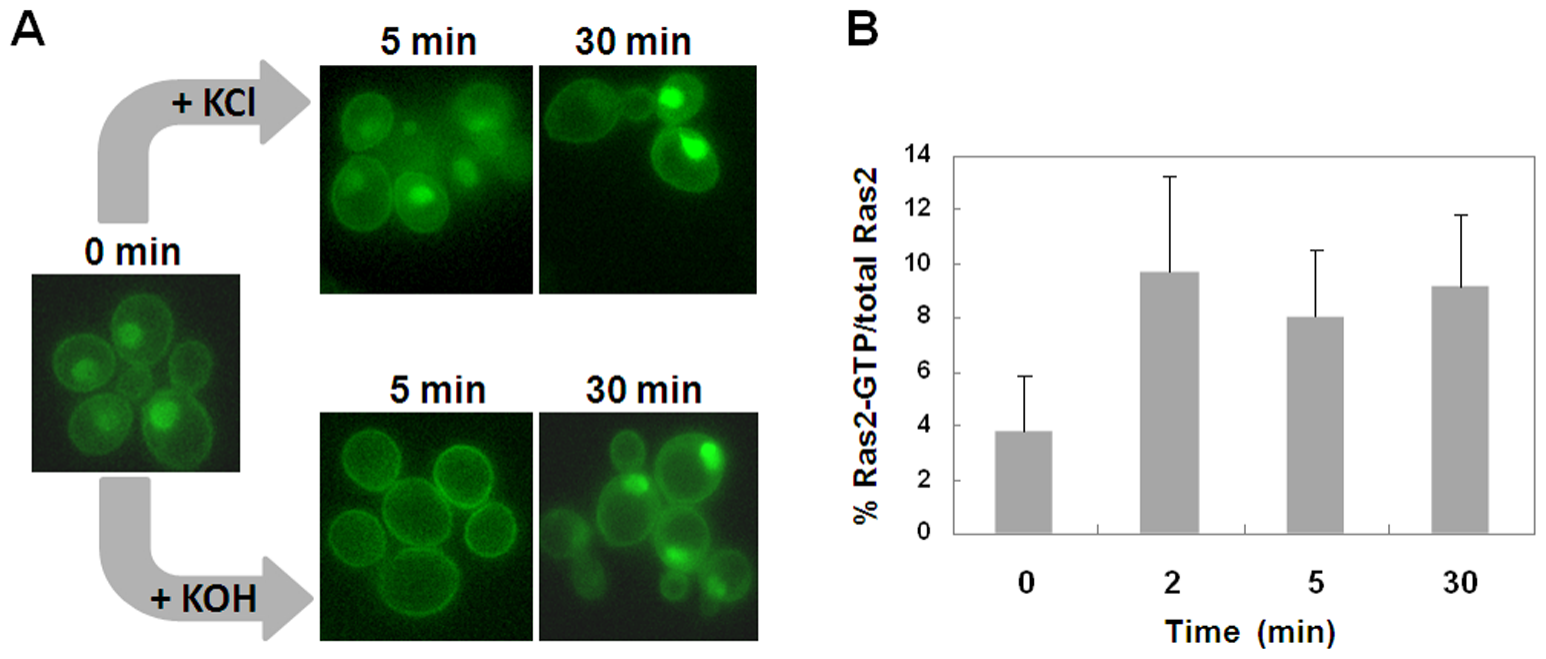
Effect of alkaline pH stress on active Ras localization and on Ras2-GTP levels in W303-1A cells. (A) W303-1A wild type cells transformed with YEpeGFP-RBD3 were grown in medium containing 2% glucose at 30°C until exponential phase. Pictures were taken, before (0 min) and after addition of either KCl (control cells) or KOH to raise the pH to 8.0. (B) Ras2-GTP/total Ras2 levels after addition of KOH to raise the pH to 8.0 to cells of the wild type W303–1A strain.

Recently it has been published that alkalinization of the medium causes a transient downregulation of the cAMP/PKA pathway [32]. In particular, the level of cAMP was measured after a shifti of the cells from pH 5.5 to medium buffered to pH 8.0 and a drastic decrease in this second messenger level was observed in the first 5–15 min, followed by a recovery to the initial levels after 30 minutes of stress. Moreover, the alkalinization also resulted in a rapid nuclear translocation of the transcriptional factor Msn2, which mediates a general stress response by binding with STRE (stress response element) sequences in the promoter of different genes [32]. To further investigate the effect of downregulation of PKA activity on active Ras proteins localization, we evaluated the effect of alkaline pH stress on the localization of these small GTPases. To this aim, the pH was raised to 8.0 by addition of KOH (12.5 mM, final concentration) directly to W303-1A wild type cells expressing the eGFP-RBD3 probe and growing exponentially in 2% glucose medium. Our results show that alkalinization caused within 5 minutes the ocalization of active Ras proteins almost exclusively to the plasma membrane, followed by a relocalization of the probe back to nuclear compartment after 30 minutes of stress (Figure 3A). The control cells received the same concentration of KCl; as expected, a nuclear and plasma membrane labelling of the cells was observed, indicating that potassium itself did not impair the proper localization of active Ras.

We used the pull-down assay to investigate whether alkaline pH stress, causing a transient downregulation of the cAMP/PKA pathway, could be related to the Ras2-GTP-loading state. Indeed our results show that addition of KOH to glucose-growing cells triggered a fast increase in the Ras2-GTP level (Figure 3B), suggesting that alkalinization downregulates the cAMP/PKA pathway acting on element(s) downstream Ras and that the increase of Ras2-GTP might be related to a decrease of the feed-back inhibition operated by PKA on Ras2 [29].

### The Nuclear Localization of Active Ras2 Protein is Required for Invasive Growth

**Figure 4:**
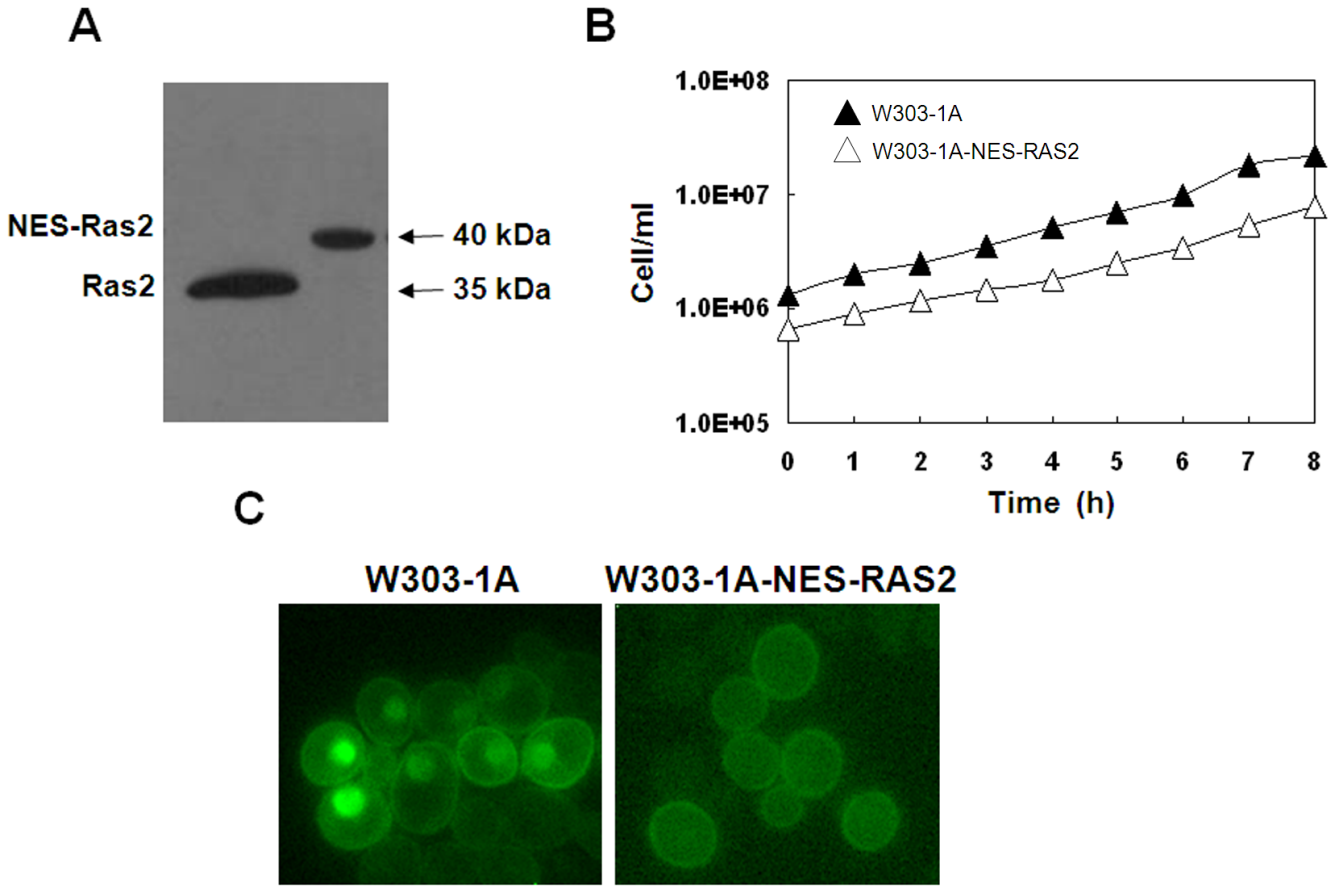
Effect of expression of NES-Ras2 on proteins level, growth rate and localization of Ras-GTP. (A) Expression of the Ras2 and NES-Ras2 proteins using a SDS-PAGE and western blotting analysis. (B) The W303-1A-NES-RAS2 strain (Δ) grew in minimal medium containing 2% glucose at a rate comparable to that of the W303-1A wild type strain (▴). (C) W303-1A and W303-1A-NES-RAS2 cells transformed with YEpeGFP-RBD3. Cells were grown in medium containing 2% glucose at 30°C until exponential phase and then photographed with a Nikon fluorescence microscope.

The eGFP-RBD3 probe was localized to the plasma membrane in wild type W303-1A cells growing exponentially on glucose medium, indicating the presence of active Ras in this cellular compartment. Surprisingly the probe was also found to accumulate within the nucleus and only marginally in mitochondria. To investigate the nuclear function of the Ras2 protein, we generated a strain where Ras2 was completely excluded from this cellular compartment. To this aim the nuclear export signal from the HIV virus (HIV virus Rev protein NES) [20] was inserted at the 5′end of the *RAS2* sequence, generating strain W303-1A-NES-RAS2. Expression of NES-Ras2 in W303-1A cells resulted in a polypeptide of the correct size (about 40 kDa) (Figure 4A) and no obvious defects in growth were observed in these cells growing in glucose containing medium (Figure 4B). As expected, the insertion of Rev NES sequence completely excluded the Ras2 protein from the nucleus (Figure 4C).

**Figure 5:**
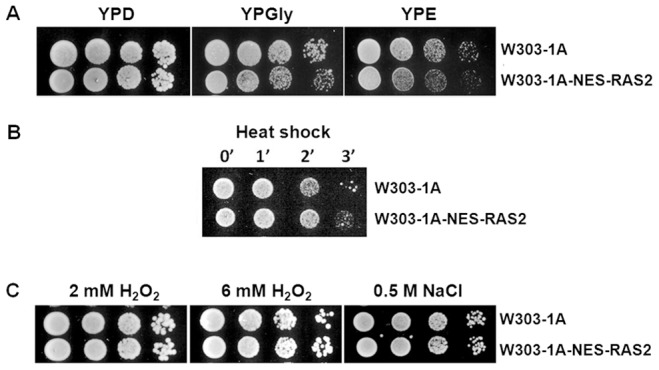
Effect of expression of NES-Ras2 on growth on different carbon sources and on PKA-activity-related phenotypes. (A) Cells were gown in YPD medium at 30°C until exponential phase. Then cells were harvested by centrifugation, washed three times with sterile water and resuspended in sterile water at 10^7^ cells/ml. 5 µl from the concentrated suspension and from 10–fold dilutions were spotted on agar plates containing the indicated carbon sources. Pictures were taken after 48 h at 30°C. (B) Heat-shock resistance in exponentially growing cells. Cells were incubated synthetic complete medium containing 2% glucose to exponential phase, diluted in fresh medium to a concentration of 1.25×10^6^ cells/ml and then exposed to heat shock at 51°C for 0, 1, 2 and 3 min. Approximately 10^4^ cells were spotted on YPD agar and incubated at 30°C for 24 hours. (C) Oxidative and osmotic stress resistance in exponentially growing cells. Cells were incubated in YPD medium until exponential phase. Then cells were harvested by centrifugation, washed three times with sterile water and resuspended in sterile water at 10^7^ cells/ml. 5 µl from the concentrated suspension and from 10–fold dilutions were spotted on glucose agar plates containing respectively 2 mM H_2_O_2_, 6 mM H_2_O_2_ and 0.5 M NaCl. After 48 hours at 30°C pictures were taken.

To characterize the nuclear function of Ras2, we first investigated the ability of W303-1A-NES-RAS2 cells to grow on media containing different carbon sources. Our results show that the mutant strain grew in YPD, YPGly and YPE media, indicating that the expression of the NES-Ras2 protein does not cause a growth defect neither on fermentable nor non-fermentable carbon sources (Figure 5A).

A possible consequence of altered localization of active Ras2 proteins could be a change of PKA activity. In order to investigate this aspect, phenotypic properties usually related to PKA activity were considered. In particular, sensitivity to heat shock, osmotic and oxidative stress have been tested. Our results show that the expression of the NES-Ras2 protein had no effect on heat shock, osmotic and oxidative stress (Figure 5B e 5C), indicating that the delocalization of active Ras2 does not influence the PKA activity related phenotypes analyzed.

**Figure 6:**
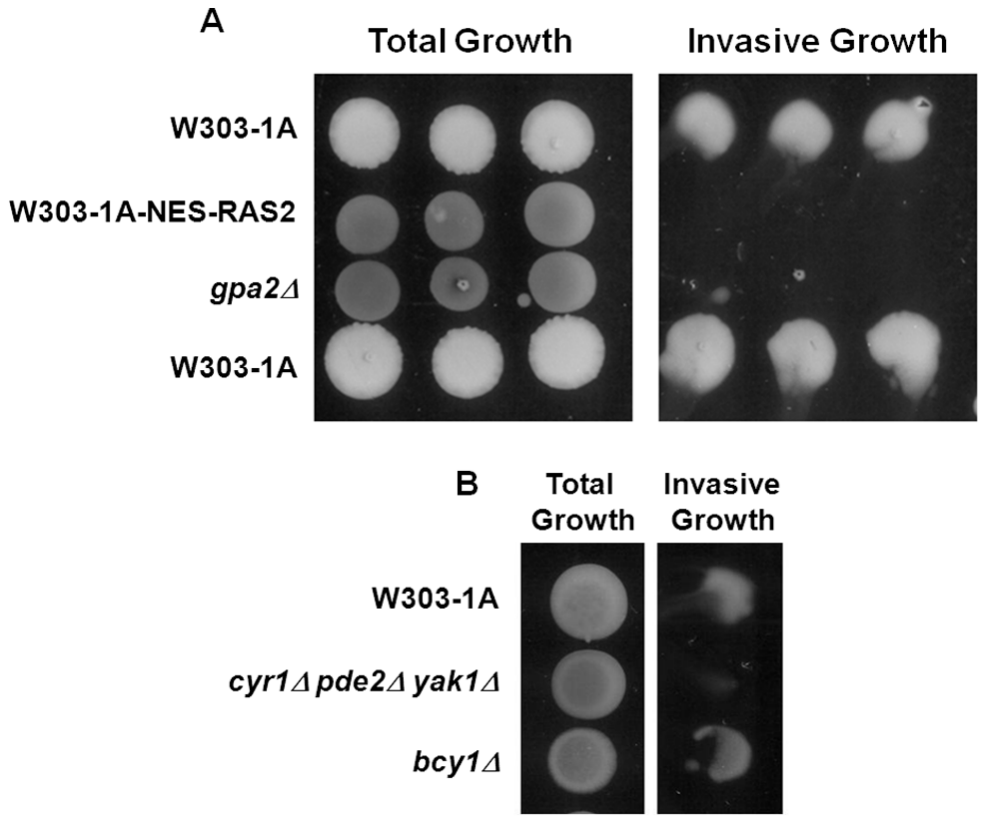
Nuclear active Ras2 is required for invasive growth in W303-1A-based strains. YPD exponentially growing cells of the indicated strains were spotted on YPD agar plates. After 3°C, the plates were gently washed with water from top down and pictures were taken before (Total growth) and after (Invasive growth) washing.

The Ras2 protein, but not Ras1, activates invasive growth using either of two downstream signalling pathways, the filamentation MAPK (Cdc42p/Ste20p/MAPK) cascade or the cAMP/PKA pathway, indicating a crosstalk between these signalling pathways and this could happen in the nucleus [16], [17], [18]. To investigate the possible involvement of nuclear active Ras2 in invasive growth, we performed an invasive growth test, including the *gpa2Δ* strain as a control strain, since disruption of Gpa2 activity was previously reported to impair invasive growth [19], [33]. As shown in Figure 6A, the weak invasive activity of the W303-1A strain was completely eliminated in *gpa2Δ* cells and in cells expressing the NES-Ras2 fusion protein, indicating that active Ras2 in the nucleus is actually required for invasive growth in this background.

Although data in literature clearly show that Ras2 activates invasive growth using either the filamentation MAPK cascade or the cAMP/PKA pathway, there are still quite some ambiguities about whether the MAPK regulates the PKA pathway or PKA regulates the MAPK pathway or the two function independently [16], [17], [18], [34]. To investigate this aspect, we performed an invasive growth test using strains with either high or absent PKA activity and we obtained data suggesting that the cAMP/PKA pathway could control the ability of the cells to perform invasive growth also by influencing the localization of active Ras proteins. In fact, as previously reported [34], we confirm that *bcy1*Δ cells, showing a predominant nuclear accumulation of active Ras, were able to invade the agar, while *cyr1*Δ *pde2*Δ *yak1*Δ cells, showing a predominant plasma membrane accumulation of active Ras, lost the invasive-growth capability, indicating that PKA activity actually controls invasive growth influencing the active Ras localization (Figure 6B).

**Figure 7:**
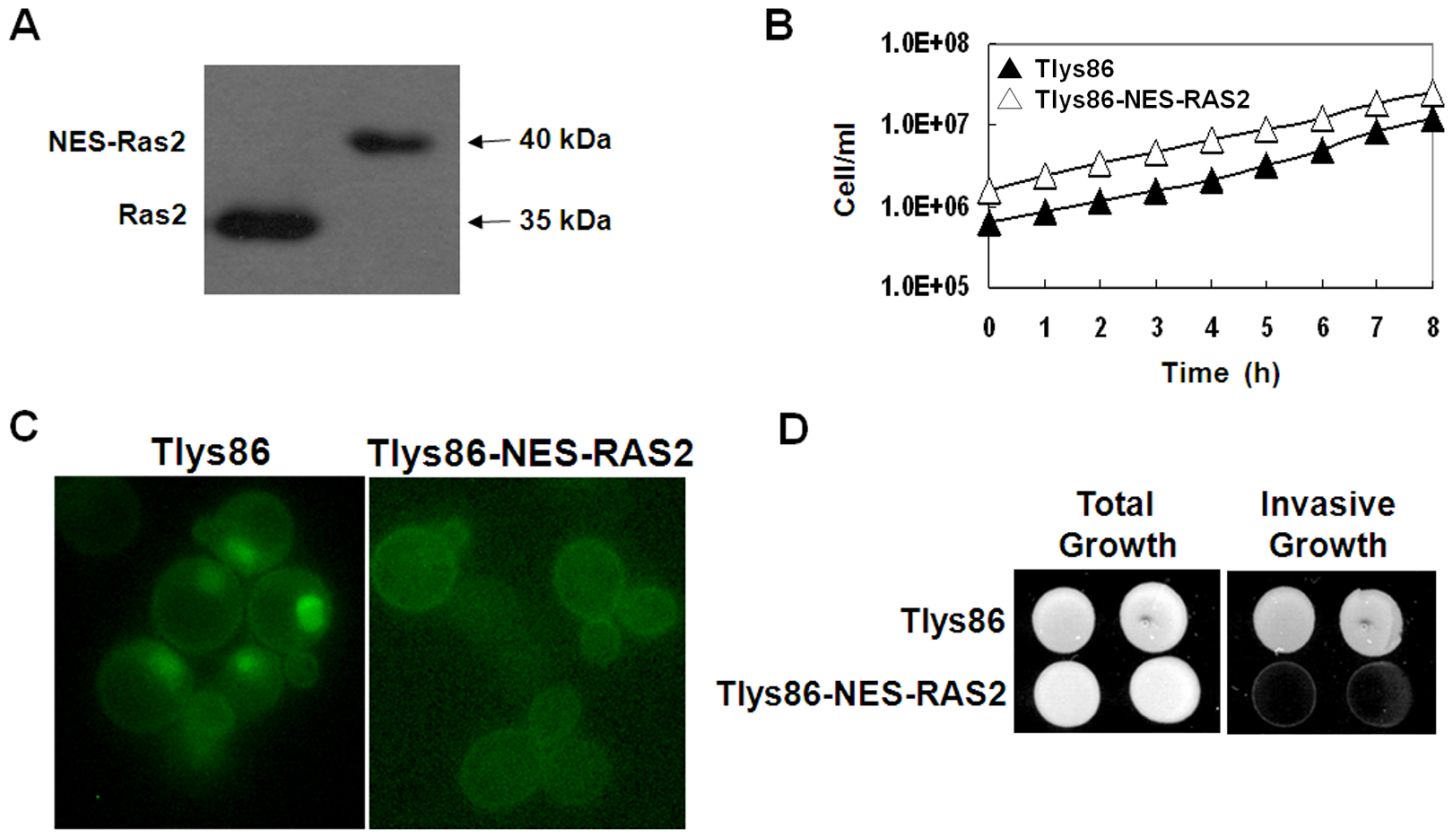
Effect of expression of NES-Ras2 on proteins level, growth rate, localization of Ras-GTP and invasive growth. (A) Expression of the Ras2 and NES-Ras2 proteins using a SDS-PAGE and western blotting analysis. (B) The Tlys86-NES-RAS2 strain (Δ) grew in minimal medium containing 2% glucose at a rate comparable to that of the Tlys86 wild type strain (▴). (C) Tlys86 and Tlys86-NES-RAS2 cells transformed with YEpeGFP-RBD3. Cells were grown in medium containing 2% glucose at 30°C until exponential phase and then photographed with a Nikon fluorescence microscope. (D) YPD exponentially growing cells of the indicated strains were spotted on YPD agar plates. After 3 days at 30°C, the plates were washed under the running water and pictures were taken before (Total growth) and after (Invasive growth) washing.

Finally to confirm the inability of cells lacking active Ras2 in the nucleus to perform invasive growth, we made a fusion between the Ras2 protein and the Rev NES sequence in the strain Tlys86 commonly used to test this phenotype [27], generating strain Tlys86-NES-RAS2. Also in this background, expression of NES-Ras2 resulted in a polypeptide of the correct size (Figure 7A), the mutant strain grew in minimal medium containing glucose at a rate comparable to that of the wild-type strain (Figure 7B) and the insertion of the Rev NES sequence completely excluded the NES-Ras2 protein from the nucleus (Figure 7C). We then performed an invasive growth test. As shown in Figure 7D, the invasive activity of the Tlys86 strain was completely eliminated in cells expressing the NES-Ras2 fusion protein, confirming that active Ras2 in the nucleus is actually required for invasive growth. We verified that, also in this background, the expression of the NES-Ras2 protein did not cause a growth defect neither on fermentable nor non-fermentable carbon sources and that the expression of the NES-Ras2 protein had no effect on heat shock, osmotic and oxidative stress (Figure S3).

## Discussion

Recently we have shown that active Ras was localized not only to the plasma membrane in wild type yeast cells growing exponentially on glucose medium, but also within the nucleus and marginally in mitochondria [13]. In this paper we show that the unexpected nuclear localization of active Ras is specific and PKA dependant. In particular, we show that active Ras was localized mainly in the nucleus in cells with high PKA activity (the *bcy1*Δ strain) or after addition of cAMP to *cyr1*Δ *pde2*Δ *yak1*Δ and *cyr1*Δ *pde2*Δ *msn2*Δ *msn4*Δ cells, while it was mainly plasma membrane-bound in cells with either low (*tpk1^w1^tpk2*Δ *tpk3*Δ) or absent PKA activity (*cyr1*Δ *pde2*Δ *yak1*Δ and *tpk1*Δ *tpk2*Δ *tpk3*Δ *yak1*Δ cells) or after alkalinization of the medium, which was previously shown to transiently decrease cAMP accumulation [32]. Moreover we show that addition of KOH to glucose-growing cells triggered a fast increase in the Ras2-GTP level, suggesting that alkalinization might influence PKA activity either in parallel or downstream Ras. Purwin *at al.* actually showed a marked pH dependence of adenylate cyclase activity [35], pointing to this enzyme to be the target of alkalinization. However, adenylate cyclase is also activated by the plasma membrane receptor Gpr1 and its associated Gα subunit Gpa2 and consequently alkalinization might regulate PKA activity through modulation of Gpa2 activity and this could happen through adenylate cyclase. Additional experiments need to be performed to test this hypothesis.

Other components of the cAMP/PKA signaling pathway are localized also in the nucleus, like Cdc25 and the Ira1 proteins [8]; [15]. Moreover, in cells growing on glucose PKA was found almost exclusively nuclear, whereas, in cells growing on non-fermentable carbon sources or in stationary phase, Bcy1 and Tpk1 were more evenly distributed over both nucleus and cytoplasm [14]. On the contrary, Cyr1 was not found in the nuclear compartment, but it was mainly localized in internal membranes [8], indicating that nuclear import could actually have the purpose to separate the protein from adenylate cyclase. Nevertheless, nuclear localization might also be indicative of a specific nuclear function of Ras-GTP. In particular, it is known that Ras2, but not Ras1, activates invasive growth using either the filamentation MAPK cascade (Cdc42p/Ste20p/MAPK) or the cAMP/PKA pathway, indicating a crosstalk between both signalling pathways and this could happen in the nucleus [16], [17], [18]. Our results show that both the W303-1A-NES-Ras2 and the Tlys86-NES-Ras2 strains, where Ras2 was excluded from the nucleus, were completely defective in invasive growth, while the expression of the NES-Ras2 protein did not interfere with PKA activity. Moreover, the involvement of active Ras in invasive growth is also substantiated by data we recently published where we show that in a strain deleted in *GPA2*, which is reported to have a defect in pseudohyphal growth [19], active Ras did not accumulate in the nucleus, but was mainly localized in mitochondria [13].

Till now, in yeast, only the adenylate cyclase protein Cyr1 has been identified as an effector protein directly binding to activated forms of Ras2, while no direct effector protein of Ras2 has been identified for stimulation of invasive growth via the Cdc42p/Ste20p/MAPK pathway. Our results suggest that nuclear active Ras2 must target effectors distinct from adenylate cyclase to activate invasive growth, since Cyr1 was not found in this cellular compartment. A possible candidate could be the Cdc42 protein, which was reported to be a regulator of filamentous growth and is likely to act downstream Ras2, since double mutants containing the activated *RAS2* allele and the dominant negative *CDC42* allele (*RAS2^Vall9^*, *CDC42^Alal18^*) fail to form filaments [36]. Moreover, this protein has been shown to colocalize with its guanine nucleotide exchange factor Cdc24 at a nuclear level. In particular, Cdc42 localizes to nuclear membranes, while Cdc24 is targeted to the nuclei [37]. In summary we can conclude that: 1) the nuclear localization of active Ras2 is required for invasive growth; 2) the exclusion of Ras2 from the nucleus does not cause a growth defect neither on fermentable nor on non-fermentable carbon sources and does not influence the PKA-activity- related-phenotypes analyzed in this work; 3) the localization of active Ras is regulated by PKA, being Ras-GTP mainly localized to the plasma membrane in strains with either low or absent PKA activity or following alkaline pH stress and predominantly nuclear in a strain with high PKA activity or following activation of the cAMP/PKA pathway by addition of cAMP to *cyr1Δ pde2Δ yak1Δ* and *cyr1Δ pde2Δ msn2Δ msn4Δ* strains; 4) PKA controls invasive growth influencing the localization of active Ras proteins. In fact, as previously reported [34], we confirm that in mutants with absent PKA activity, active Ras is plasma membrane localized and cells can not invade the agar, whereas invasiveness is restored in cells showing high PKA activity and a nuclear localization of active Ras.

## Supporting Information

Figure S1 **Effect of PKA activity on the localization of active Ras.** (A) SP1, *tpk1w1 tpk2*Δ *tpk3*Δ and *tpk1*Δ *tpk2*Δ *tpk3*Δ *yak1*Δ cells transformed with YEpeGFP-RBD3. Cells were grown in medium containing 2% glucose at 30°C until exponential phase and then photographed with a Nikon fluorescence microscope. (B) Subcellular distribution of eGFP fluorescence.
(TIF)

Figure S2 **Localization of active Ras in glucose-growing** ***cyr1Δ pde2Δ msn2Δ msn4Δ*** **cells, before and after addition of cAMP.** (A) *cyr1Δ pde2Δ msn2Δ msn4Δ* cells transformed with YEpeGFP-RBD3 were grown in medium containing 2% glucose at 30°C until exponential phase and then photographed with a Nikon fluorescence microscope, before and 45 min after addition of 2 mM cAMP. (B) Subcellular distribution of eGFP fluorescence.
(TIF)

Figure S3 **Effect of expression of NES-Ras2 on growth on different carbon sources and on PKA-activity-related phenotypes.** (A) Cells were gown in YPD medium at 30°C until exponential phase. Then cells were harvested by centrifugation, washed three times with sterile water and resuspended in sterile water at 10^7^ cells/ml. 5 µl from the concentrated suspension and from 10–fold dilutions were spotted on agar plates containing the indicated carbon sources. Pictures were taken after 48 h at 30°C. (B) Heat-shock resistance in exponentially growing cells. Cells were incubated synthetic complete medium containing 2% glucose to exponential phase, diluted in fresh medium to a concentration of 1.25×10^6^ cells/ml and then exposed to heat shock at 51°C for 0, 1, 2 and 3 min. Approximately 104 cells were spotted on YPD agar and incubated at 30°C for 3 days. (C) Oxidative and osmotic stress resistance in exponentially growing cells. Cells were incubated in YPD medium until exponential phase. Then cells were harvested by centrifugation, washed three times with sterile water and resuspended in sterile water at 10^7^ cells/ml. 5 µl from the concentrated suspension and from 10–fold dilutions were spotted on glucose agar plates containing respectively 2 mM H_2_O_2_, 6 mM H_2_O_2_ and 0.5 M NaCl. After 48 hours at 30°C pictures were taken.
(TIF)
