# Supplementary figures and images for: Nuclear Ras2-GTP Controls Invasive Growth in Saccharomyces cerevisiae
Source: PLoS One. 2013 Nov 14;8(11):e79274. doi: 10.1371/journal.pone.0079274 (PMC3828362; doi:10.1371/journal.pone.0079274)

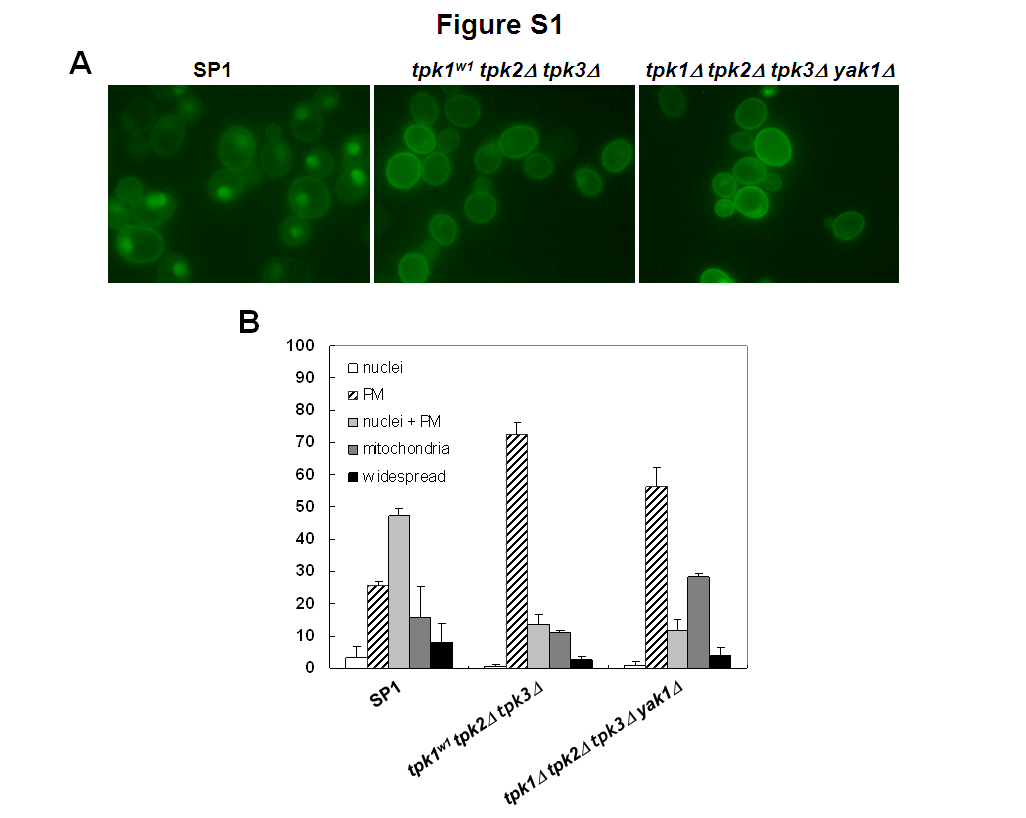

Supplement: Figure S1 — Effect of PKA activity on the localization of active Ras. (A) SP1, tpk1w1 tpk2Δ tpk3Δ and tpk1Δ tpk2Δ tpk3Δ yak1Δ cells transformed with YEpeGFP-RBD3. Cells were grown in medium containing 2% glucose at 30°C until exponential phase and then photographed with a Nikon fluorescence microscope. (B) Subcellular distribution of eGFP fluorescence. (TIF) [file pone.0079274.s001.tif]

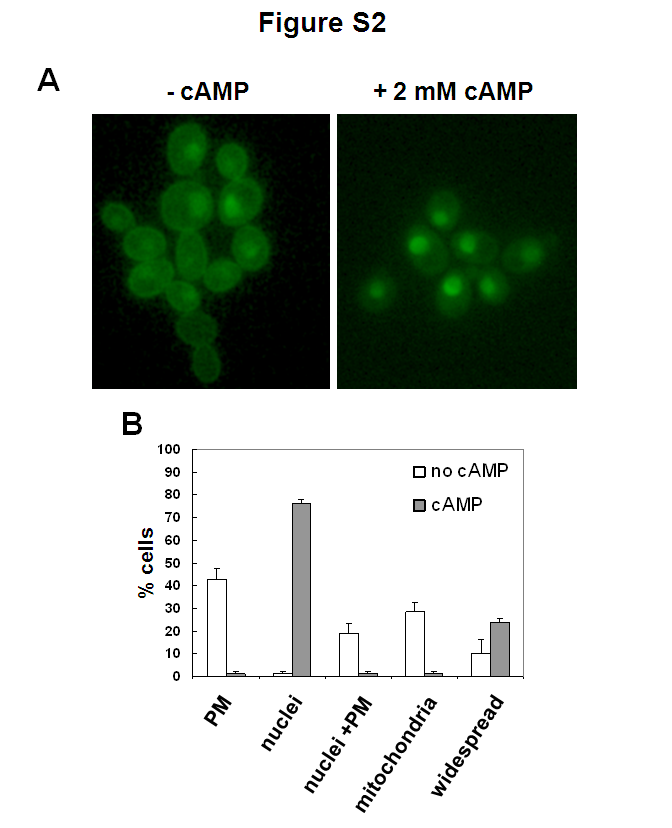

Supplement: Figure S2 — Localization of active Ras in glucose-growing cyr1Δ pde2Δ msn2Δ msn4Δ cells, before and after addition of cAMP. (A) cyr1Δ pde2Δ msn2Δ msn4Δ cells transformed with YEpeGFP-RBD3 were grown in medium containing 2% glucose at 30°C until exponential phase and then photographed with a Nikon fluorescence microscope, before and 45 min after addition of 2 mM cAMP. (B) Subcellular distribution of eGFP fluorescence. (TIF) [file pone.0079274.s002.tif]

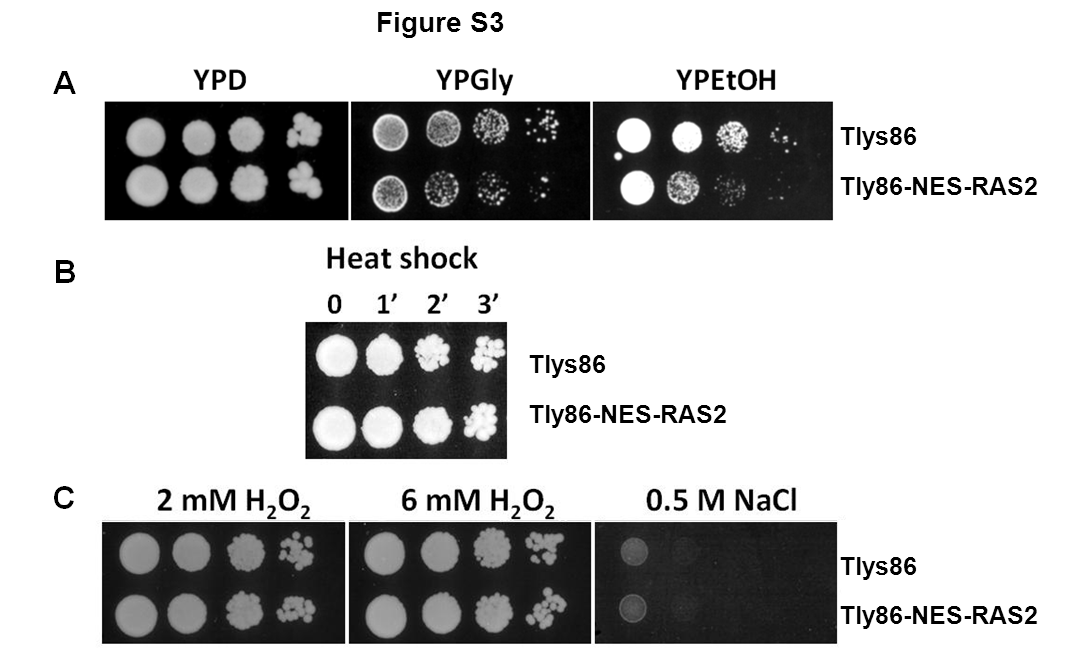

Supplement: Figure S3 — Effect of expression of NES-Ras2 on growth on different carbon sources and on PKA-activity-related phenotypes. (A) Cells were gown in YPD medium at 30°C until exponential phase. Then cells were harvested by centrifugation, washed three times with sterile water and resuspended in sterile water at 107 cells/ml. 5 µl from the concentrated suspension and from 10–fold dilutions were spotted on agar plates containing the indicated carbon sources. Pictures were taken after 48 h at 30°C. (B) Heat-shock resistance in exponentially growing cells. Cells were incubated synthetic complete medium containing 2% glucose to exponential phase, diluted in fresh medium to a concentration of 1.25×10^6^ cells/ml and then exposed to heat shock at 51°C for 0, 1, 2 and 3 min. Approximately 104 cells were spotted on YPD agar and incubated at 30°C for 3 days. (C) Oxidative and osmotic stress resistance in exponentially growing cells. Cells were incubated in YPD medium until exponential phase. Then cells were harvested by centrifugation, washed three times with sterile water and resuspended in sterile water at 107 cells/ml. 5 µl from the concentrated suspension and from 10–fold dilutions were spotted on glucose agar plates containing respectively 2 mM H2O2, 6 mM H2O2 and 0.5 M NaCl. After 48 hours at 30°C pictures were taken. (TIF) [file pone.0079274.s003.tif]
